# Discrimination of *Escherichia coli* O157, O26 and O111 from Other Serovars by MALDI-TOF MS Based on the *S10*-GERMS Method

**DOI:** 10.1371/journal.pone.0113458

**Published:** 2014-11-20

**Authors:** Teruyo Ojima-Kato, Naomi Yamamoto, Mayumi Suzuki, Tomohiro Fukunaga, Hiroto Tamura

**Affiliations:** 1 Hub of Knowledge Aichi, Aichi Science and Technology Foundation, Yakusa, Toyota, Aichi, Japan; 2 School of Agriculture, Meijo University, Shiogamaguchi, Tenpaku-ku, Nagoya, Aichi, Japan; 3 Japan Food Research Laboratories, Osu, Naka-ku, Nagoya, Aichi, Japan; Animal Health and Veterinary Laboratories Agency, United Kingdom

## Abstract

Enterohemorrhagic *Escherichia coli* (EHEC), causes a potentially life-threatening infection in humans worldwide. Serovar O157:H7, and to a lesser extent serovars O26 and O111, are the most commonly reported EHEC serovars responsible for a large number of outbreaks. We have established a rapid discrimination method for *E. coli* serovars O157, O26 and O111 from other *E. coli* serovars, based on the pattern matching of mass spectrometry (MS) differences and the presence/absence of biomarker proteins detected in matrix-assisted laser desorption/ionization time-of-flight MS (MALDI-TOF MS). Three biomarkers, ribosomal proteins S15 and L25, and acid stress chaperone HdeB, with MS *m*/*z* peaks at 10138.6/10166.6, 10676.4/10694.4 and 9066.2, respectively, were identified as effective biomarkers for O157 discrimination. To distinguish serovars O26 and O111 from the others, DNA-binding protein H-NS, with an MS peak at *m*/*z* 15409.4/15425.4 was identified. Sequence analysis of the O157 biomarkers revealed that amino acid changes: Q80R in S15, M50I in L25 and one mutation within the start codon ATG to ATA in the encoded HdeB protein, contributed to the specific peak pattern in O157. We demonstrated semi-automated pattern matching using these biomarkers and successfully discriminated total 57 O157 strains, 20 O26 strains and 6 O111 strains with 100% reliability by conventional MALDI-TOF MS analysis, regardless of the sample conditions. Our simple strategy, based on the *S10-spc-alpha* operon gene-encoded ribosomal protein mass spectrum (*S10*-GERMS) method, therefore allows for the rapid and reliable detection of this pathogen and may prove to be an invaluable tool both clinically and in the food industry.

## Introduction

Matrix-assisted laser desorption/ionization time-of-flight mass spectrometry (MALDI-TOF MS) is a robust approach for the rapid identification of microorganisms. The identification mechanism is based on the protein MS pattern obtained by MALDI-TOF MS matching microbial sequence data in available databases, the so-called fingerprinting method. This method has been rapidly developed and expanded, and has been successfully applied to the clinical field because it offers a stable, rapid and cost-effective system for microbial identification.

In using MALDI-TOF MS for the identification of microorganisms, the majority of the high-intensity MS peaks detected is derived from ribosomal proteins encoded in the *S10-spc-alpha* operon, where at least half of the ribosomal subunit proteins are encoded. This operon is highly conserved among eubacterial genomes [1]–[5]. These peaks can be reliable biomarkers with which to discriminate bacteria at a strain or pathovar level because strain-specific peaks can be predicted and verified from the DNA sequence information before measurement [6], [7]. This methodology, known as the ‘*S10*-GERMS (*S10-spc-alpha* operon gene-encoded ribosomal protein mass spectrum) method’, offers theoretically calculated *m/z* ion peaks of ribosomal proteins that are species- or strain-specific. An accurate database can then be constructed by comparing the experimentally observed *m/z* values with the theoretical values. The *S10*-GERMS method has been effectively employed in the identification of serovars of *Pseudomonas syringae* [7] and strains of *Lactobacillus casei* [8]. Strain typing by direct bacterial profiling has increasingly been studied as a method for bacterial species identification in recent years [9]–[11].

Shiga toxin-producing *Escherichia coli*, known as enterohemorrhagic *E. coli* (EHEC), causes bloody diarrhea, hemorrhagic colitis and life-threatening hemolytic-uremic syndrome. Serovar O157:H7 is the most commonly reported EHEC serovar causing many outbreaks and significantly threatening human life worldwide. Serovars O26 and O111 are also responsible for a large number of EHEC outbreaks.

Attempts to classify EHEC serovars by MALDI-TOF MS have been reported [12]; however, the results are dependent on sample preparation conditions and the biomarker proteins are not assigned. To allow this method to be practically applied in the field, it needs to be versatile and reliable. In another study, *E. coli* O157:H7-specific biomarkers HdeA, HdeB, CspC, YbgS, YjbJ and YbgO were identified using MALDI-TOF/TOF-MS/MS [13], in which only 1 Da difference was sufficient to distinguish *E. coli* O157 from other serovars.

Here, we report the discrimination of *E. coli* O157, O26 and O111 serovars with four specific biomarker proteins based on the *S10*-GERMS method by MALDI-TOF MS. These biomarker peaks that are assigned and validated by DNA sequence analysis are detected under any of the sample conditions tested, with high reproducibility, using conventional MALDI-TOF MS analysis.

## Materials and Methods

### Bacterial strains and growth conditions

Thirty EHEC strains, 4 shiga toxin non-producing O157 strains and 16 non-EHEC strains were used for the construction of a theoretical mass database (Table 1). They were purchased from the National BioResource Project (NBRP; a division of pathogenic microbe, Gifu University, Gifu, Japan), the American Type Culture Collection (ATCC; Rockville, MD, USA), the Japan Collection of Microorganisms, RIKEN BRC (JCM, Tsukuba, Japan), which is participating in the National BioResource Project of the Ministry of Education, Culture, Sports, Science and Technology, Japan, and the Biological Resource Center at the National Institute of Technology and Evaluation (NITE, Kisarazu, Japan). Three shiga toxin non-producing O157 (strains WT-141, WT-351 and WT-352) were kindly provided by Dr. Hiroshi Asakura (National Institute of Health Sciences, Japan). Nutrient broth (Becton Dickinson, Franklin Lakes, NJ, USA), tryptone soya agar (Thermo Scientific, Waltham, MA), or Luria–Bertani broth (Nacalai, Kyoto, Japan) were used for cultivation. For the blind test, another 57 *E. coli* strains, namely 12 *E. coli* strains (strains jfrl 01–12), that were isolated from food samples through 1996 to 2010 and identified as O157 or O26 by the antisera coagglutination test (Denka Seiken, Tokyo, Japan), and 45 *E. coli* strains that were kindly provided from Aichi Prefectural Institute of Public Health (APIPH) were used (Table 1). The production of verotoxin in these strains was also checked by the coagglutination test (VTEC-RPLA, Denka Seiken).

**Table 1:** *E. coli* strains used in this study.

Strain	Characteristics	Source
GTC 03904	O157:H7, VT–Shiga toxin negative	NBRP
GTC 14513	O157:H7:VT2	NBRP
GTC 14535	O157:H7:VT1&2	NBRP
GTC 14536	O157:H7:VT1&2	NBRP
GTC 14537	O157:H7:VT2	NBRP
GTC 14544	O157:H7:VT1&2	NBRP
GTC 14545	O157:H7:VT1&2	NBRP
GTC 14546	O157:H7:VT2	NBRP
GTC 14547	O157:H7:VT2	NBRP
GTC 14550	O157:H7:VT2	NBRP
GTC 14551	O157:H7:VT1&2	NBRP
GTC 14552	O157:H7:VT1&2	NBRP
GTC 14553	O157:H7:VT2	NBRP
GTC 14507	O111:H-:VT1&2	NBRP
GTC 14517	O111:H-:VT1	NBRP
GTC 14515	O26:H11:VT1&2	NBRP
GTC 14516	O26:H11:VT1	NBRP
GTC 14538	O26:H-:VT1	NBRP
GTC 14539	O26:H11:VT1&2	NBRP
GTC 14540	O26:H11:VT1	NBRP
GTC 14548	O26:H-:VT1	NBRP
GTC 14549	O26:H11:VT1	NBRP
GTC 14557	O26:H11:VT1	NBRP
GTC 14558	O26:H11:VT1	NBRP
GTC 14530	O121:H19:VT2	NBRP
GTC 14601	O121:H19:VT2	NBRP
GTC 14602	O121:H19:VT2	NBRP
GTC 14518	O115:H10:VT1	NBRP
GTC 14529	O119:H2:VT1	NBRP
GTC 14559	O63:H6:VT2	NBRP
GTC 14603	O128:H-:VT1&2	NBRP
NBRC 12713	Genome sequenced K-12 strain. The alias of W3110.	NITE
ATCC 47076	Genome sequenced K-12 strain. The alias of MG1655.	ATCC
NBRC 13893		NITE
NBRC 15034		NITE
NBRC 14237		NITE
NBRC 13891		NITE
NBRC 3301	K-12 strain.	NITE
NBRC 3972		NITE
NBRC 12062		NITE
NBRC 13168		NITE
NBRC 3548		NITE
NBRC 12734		NITE
JCM16574	Genome sequenced strain, O152:H28	JCM
ATCC BAA-1743	Genome sequenced strain	ATCC
JCM16575	Genome sequenced strain, O150:H5	JCM
NBRC 3991		NITE
WT-141	O157: VT- Shiga toxin Negative, isolated from human	
WT-351	O157: VT- Shiga toxin Negative, isolated from cattle	
WT-352	O157: VT- Shiga toxin Negative, isolated from cattle	
jfrl 01	O157:H7:VT2, isolated from pork in 1998	
jfrl 02	O157:H7:VT2, isolated from beef in 1996	
jfrl 03	O157:H7:VT1&2, isolated from beef in 1998	
jfrl 04	O157:H7:VT1&2, isolated from beef in 1996	
jfrl 05	O157:H7:VT2, isolated from welsh onion in 1996	
jfrl 06	O157:VT1&2, isolated from beef in 2003	
jfrl 07	O157:VT 2, isolated from beef in 1999	
jfrl 08	O157: VT1&2, isolated from beef in 1999	
jfrl 09	O157:VT2, isolated from beef in 2010	
jfrl 10	O157:VT2, isolated from beef in 2010	
jfrl 11	O157:VT2, isolated from beef in 2010	
jfrl 12	O26, VT1, isolated from beef in 2010	
A11-1	O157:H7, VT1&2	APIPH
A11-85	O157:HUT, VT1&2	APIPH
A11-87	O157:H7, VT1&2	APIPH
A11-88	O157:H7, VT1&2	APIPH
A11-89	O157:H7, VT1&2	APIPH
A11-90	O157:H7, VT1&2	APIPH
A11-161	O157:H7, VT2	APIPH
A11-163	O157:H7, VT2	APIPH
A11-168	O157:H7, VT1	APIPH
A11-169	O157:H7, VT1&2	APIPH
A11-175	O157:H7, VT1	APIPH
A11-176	O157:H7, VT1	APIPH
A11-177	O157:H7, VT1	APIPH
A11-225	O157:H7, VT2	APIPH
A11-234	O157:H7, VT2	APIPH
A12-154	O157:H7, VT2	APIPH
A12-163	O157:H7, VT1&2	APIPH
A12-164	O157:H7, VT2	APIPH
A12-166	O157:H7, VT1&2	APIPH
A12-167	O157:H7, VT1&2	APIPH
A12-183	O157:H7, VT2	APIPH
A12-185	O157:HUT, VT1&2	APIPH
A12-190	O157:H7, VT1&2	APIPH
A12-191	O157:HUT, VT1&2	APIPH
A12-193	O157:HUT, VT1&2	APIPH
A12-201	O157:H7, VT2	APIPH
A12-209	O157:H7, VT2	APIPH
A12-212	O157:H7, VT1&2	APIPH
A12-222	O157:H7, VT2	APIPH
A12-223	O157:H7, VT2	APIPH
A12-97	O26:H11, VT1	APIPH
A12-98	O26:H11, VT1	APIPH
A12-99	O26:H11, VT1	APIPH
A12-100	O26:H11, VT1	APIPH
A12-147	O26:H11, VT1	APIPH
A13-137	O26:H11, VT1	APIPH
A13-138	O26:H11, VT1	APIPH
A13-154	O26:H11, VT2	APIPH
A13-155	O26:H11, VT2	APIPH
A13-165	O26:H11, VT1	APIPH
A12-152	O111:HUT, VT1	APIPH
A12-161	O111:H21, VT1	APIPH
A12-162	O111:H21, VT1&2	APIPH
A12-200	O111:HUT, VT1	APIPH
A12-216	O121:H19, VT2	APIPH

### Construction of the protein mass database

The amino acid sequences of ribosomal subunit proteins and biomarker candidates of genome sequenced strains were obtained from the National Center for Biotechnology Information (NCBI) database. The theoretical ionized mass of each protein was calculated using a Compute pI/Mw tool on the ExPASy proteomics server (http://web.expasy.org/compute_pi/), considering the N-terminal rule. For the non-genome-sequenced strains, the DNA sequence of the ribosomal proteins encoded in the *S10-spc-alpha* operon and biomarker candidates were analyzed as described previously [14]. In brief, respective regions of ribosomal protein-encoding genes (≈5 kbp) or genes encoding biomarker proteins were amplified using high-fidelity DNA polymerase, KOD plus (Toyobo, Osaka, Japan), and primers designed against the consensus DNA sequences up- and down-stream of the target regions in the *E. coli* genome sequences in the NCBI database. Sequencing reactions were carried out using a BigDye ver. 3.1 Cycle Sequencing Kit (Applied Biosystems, Foster City, CA, USA). DNA primers used for PCR and sequence analysis are listed in Table 2.

**Table 2:** Primers used in this study.

Name	Sequence (5′ – 3′)	Purpose
EcW3110-S10-F	AAGAACGGTTACACTCTCCC	amplification of *S10* region
EcW3110-S10-R	ACACCGCTTCAAGGATATGG	amplification of *S10* region
EcW3110-S10-1	AATCGTAATGGGTCTGAGGAG	sequencing
EcW3110-S10-2	AAGCTGGCCACTTCGCTAAAG	sequencing
EcW3110-S10-3	TGCTGAAGTAACTGGTTCCGG	sequencing
EcW3110-S10-4	AAGCTGCTGTGCAGAAACTG	sequencing
EcW3110-S10-5	CATAACGTAGAAATGAAACCAGG	sequencing
EcW3110-S10-6	ACGTTCCGGTATTTGTAACCG	sequencing
EcW3110-S10-7	TCAGTACCTGACTAAGGAAC	sequencing
EcW3110-S10-8	AGCGTCGCTGATGTTACAAC	sequencing
EcW3110-S10-9	AGCAAGTGCGTCGCGATGTCG	sequencing
EcW3110-S10-10	GCTGGCATGATTCGTGAAGAACG	sequencing
EcW3110-spc-F	AACGGCTCAGAAATGAGCCG	amplification of *spc* region
EcW3110-spc-R	AGCAGTCTGCGTTTCAGCTC	amplification of *spc* region
EcW3110-spc-1	TCTACCCATATCCTTGAAGC	sequencing
EcW3110-spc-2	ATTGTTGAAGGTATCAACCTG	sequencing
EcW3110-spc-3	TCGTGGTAACTACAGCATG	sequencing
EcW3110-spc-4	ACCATGCCTTCCTCCAAGCT	sequencing
EcW3110-spc-5	TTGGTGTAGGTTACCGTGCAG	sequencing
EcW3110-spc-6	ATGCTGCCCGTGAAGCTGGC	sequencing
EcW3110-spc-7	ATCGGTCGTCTGCCGAAACAC	sequencing
EcW3110-spc-9	GTCACCATGCCTTCCTCCAAG	sequencing
EcW3110-spc-1r	GATGATGTCGCCTACGCCTGC	sequencing
EcW3110-spc-2r	TTACCGGTTAACACGATAAC	sequencing
EcW3110-alpha-F	AGTGCCAAAGGTGGCTTAGGC	amplification of *alpha* region
EcW3110-alpha-R	ACAGCTATTGTAGATAAGTGG	amplification of *alpha* region
EcW3110-alpha-1	TGCCCATACTATCGAGCAAGC	sequencing
EcW3110-alpha-2	TCACTGCTTATCGTTGTTGTC	sequencing
EcW3110-alpha-3	TGTCGTTGAAGGTGATCTGCG	sequencing
EcW3110-alpha-4	AATGGCAAGATATTTGGGTC	sequencing
EcW3110-alpha-5	TGCGGACATTAACGAACACCTG	sequencing
EcW3110-alpha-6	TGCCTACAATGTTGAAGCAGCG	sequencing
EcW3110-alpha-7	AGCTGCGCCGCGTAGTTGAGC	sequencing
EcW3110-alpha-1r	AGCTGGATAATGATCGACGC	sequencing
EcW3110-L25-F	TTCGAGCAGCTTTTTATCCGCC	amplification of L25
EcW3110-L25-R	AAGGCTACGAACTGGAAGAGAGC	amplification of L25
EcW3110-L25-1	ATACGCGCACACCGGGCATC	sequencing
EcW3110-L25-1r	AGACCGTAGCACACTGCGTCAG	sequencing
EcW3110-S15-F	TACGAACGATCGGATTAAGCAATG	amplification of S15
EcW3110-S15-R	TTACTTGATCCATTACTGATGCC	amplification of S15
EcW3110-S15-1	GGATTAAGCAATGTAATATCC	sequencing
EcW3110-S15-1r	ATTACTGATGCCAATGGACAGTCC	sequencing
Ec_HdeB-F	GATATGTAATTCCGGGAATGC	amplification and sequencing of HdeB
Ec_HdeB-R	AAGGAGCAGCAAGATGGCTCAAC	amplification of HdeB
Ec_YdaQ-F	TCATAGCTGATTATTAATAATC	amplification of YdaQ
Ec_YdaQ-R	ATGAACCAGATGCGAATGTAT	amplification of YdaQ
Ec_HNS-F	TGAATTCCTTACATTCCTGGC	amplification and sequencing of H-NS
Ec_HNS-R	AGCTTATTCTTATTAAATTGTC	amplification of H-NS

### MALDI-TOF MS analysis for the evaluation of the mass database

Bacterial colonies grown on agar plate were picked and placed directly onto a measurement steel plate, while bacteria from liquid culture were harvested by centrifugation then washed with TMA-I buffer (10 mM Tris-HCl pH 7.8, 30 mM NH_4_Cl, 10 mM MgCl_2_ and 6 mM 2-mercaptoethnol). Approximately 10^7^ cfu were mixed well with 1 µL of matrix solution consisting of 20 mg/mL sinapic acid (Wako Pure Chemical) or saturated α-cyano-4-hydroxycinnamic acid (CHCA), and 1% (v/v) trifluoroacetic acid (Wako Pure Chemical) in 50% (v/v) acetonitrile. The mixture was spotted onto the MALDI sample plate and air dried. MALDI-TOF MS analysis was performed using an AXIMA micro-organism identification (Shimadzu/Kratos, Kyoto, Japan) as described previously, with minor modifications [7]. Briefly, the sample was measured in the positive linear mode in the spectrum range of *m*/*z* 2000–20000. Data were obtained from the sum of 100 individual laser shots and calibrated with the *E. coli* strain DH5α using the peaks at *m/z* 4365.4, 7274.5, 10300.1, 12770.6 and 14365.6, corresponding to ribosomal proteins L36, L29, S19, L18 and L17, respectively. After calibrating manually, each sample was automatically calibrated with the same internal peaks as DH5α. Theoretical and measured masses were matched with 500 ppm tolerance. The actual masses in the MALDI-TOF MS spectra were matched with the theoretical values and corrected appropriately.

### Automated MALDI-TOF MS analysis for validation

Samples prepared from colonies were automatically analyzed to verify the effectiveness and reproducibility of selected biomarkers. Four analytes per strain were prepared as described above. To evaluate the effects of culture medium on the masses of selected biomarkers, typical selective media for *Enterobacteriaceae* or O157, desoxycholate agar (Nissui Pharmaceutical, Tokyo, Japan), CT-SMAC (Kyokuto Pharmaceutical Industrial, Tokyo, Japan), Chromagar X-gal (Chromagar, Paris, France), and crystal violet neutral red bile lactose agar (VRBL, Thermo scientific) were tested in addition to the normal growth media such as nutrient broth, tryptone soya agar or Luria–Bertani broth.

### Cluster analysis

Fingerprints of protein mass patterns were analyzed with SARAMIS (Spectral Archive and Microbial Identification System, AnagnosTec, Postdam-Golm, Germany) to construct binary matrices of biomarkers. The data were imported into the PAST software (http://folk.uio.no/ohammer/past/, Natural History Museum, Oslo University, Norway) to calculate distance matrices using the neighbor-joining method with Kimura algorithm. A phylogenetic tree was constructed using the FigTree ver. 1.4.0 software (http://tree.bio.ed.ac.uk/software/figtree/) as described previously [16].

### Blind test using isolated wile-type *E. coli* strains

To evaluate the discrimination method using our selected biomarkers, 57 *E. coli* strains, individually isolated from food (such as beef, pork and Welsh onions) or humans and identified as serovars O157, O26, O111 or O121 by antisera testing, were analyzed by MALDI-TOF MS. Semi-automated classification was demonstrated according to the mass patterns of selected four biomarker proteins.

### Nucleotide sequence accession numbers

The nucleotide sequences of ribosomal proteins encoded in the *S10-spc-alpha* operon, biomarker proteins S15 and L25, acid stress chaperon HdeB and DNA-binding protein H-NS, of *E. coli* strains determined in this study were deposited in the DNA data bank of Japan (DDBJ, http://www.ddbj.nig.ac.jp) with accession numbers from AB903039 to AB903902 and AB915955 to AB916334.

## Results and Discussion

### Construction of the protein mass database

In this study we have attempted to employ the *S10*-GERMS method for the discrimination of major serovars of EHEC O157, O26 and O111 from the others. The theoretical masses of ribosomal proteins encoded by the *S10-spc-alpha* operon were calculated based on the sequence analysis and genome sequence information (Table 3). The mass values were compared with the actual analytical results of MALDI-TOF MS and manually validated. The masses of the *S10-spc-alpha* operon-encoded ribosomal proteins not shown in Table 3, namely S10, L3, L4, L23, L2, S19, L22, S3, L16, L29, S17, L14, L5, S14, S8, L6, L18, S5, L30, L36, S13, S11, S4 and L17, were all identical respectively in all of the *E. coli* strains used for database construction. Whereas, L24, S5 and S13, thought to be biomarker candidates from their calculated masses, gave unclear peaks because of small differences in masses or high molecular weights (Table 3). The *S10*-GERMS method has successfully been employed for *Pseudomonas* sp., *Bacillus* sp. and *Lactobacillus* sp. in previous studies [7], [8], [14], [17]. However, in the case of *E. coli*, strain or serovar typing using ribosomal proteins encoded in the *S10-spc-alpha* operon appears to be more challenging due to a less diversity of the masses. Although the ribosomal proteins encoded in the *S10-spc-alpha* operon were not suitable as biomarkers for serovars O157, O26 and O111, the other strains which are classified into group G to P in Table 1 show unique mass patterns of ribosomal proteins in the operon. It helps the strain level discrimination of *E. coli* using these biomarkers.

**Table 3:** Theoretical masses of selected biomarker proteins for *E. coli* discrimination.

		Group of mass pattern															
		A	B	C	D	E	F	G	H	I	J	K	L	M	N	O	P
Protein	Coded operon	O157	O157	O157	O26 O111	O26	O121, O128, O152, -	O115	O119	O63	K12	-	-	-	-	-	O150
L23	S10	11200.1	11200.1	11200.1	11200.1	11200.1	11200.1	11147.1	11200.1	11200.1	11200.1	11200.1	11200.1	11200.1	11200.1	11200.1	11200.1
L24	spc	11186.0	11186.0	11186.0	11186.0	11186.0	11186.0	11186.0	11186.0	11216.0	11186.0	11186.0	11216.0	11186.0	11186.0	11186.0	11216.0
S14	spc	11450.3	11450.3	11450.3	11450.3	11450.3	11450.3	11450.3	11450.3	11450.3	11450.3	11450.3	11450.3	11450.3	11464.3	11450.3	11450.3
L15	spc	14967.4	14967.4	14967.4	14967.4	14967.4	14967.4	14981.4	14945.0	14967.4	14981.4	14981.4	14967.4	14981.4	14967.4	14967.4	14967.4
S11+Me	alpha	13728.8	13728.8	13728.8	13728.8	13728.8	13728.8	13728.8	13728.8	13728.8	13728.8	13728.8	13728.8	13728.8	13728.8	13728.8	13756.8
YdaQ		8325.6	-	8325.6	8325.6	-	8325.6	8325.6	8325.6	8325.6	8325.6	8325.6	-	-	8325.6	-	-
S15		**10166.6**	**10166.6**	10138.6	10138.6	10138.6	10138.6	10138.6	10138.6	10138.6	10138.6	10137.6	10138.6	10138.6	10138.6	10138.6	10138.6
L25		**10676.4**	**10676.4**	10694.4	10694.4	10694.4	10694.4	10694.4	10694.4	10693.5	10694.4	10694.4	10693.5	10694.4	10694.4	10693.5	10693.5
HdeB		-	-	-	9066.2	9066.2	9066.2	9066.2	9066.2	9066.2	9066.2	9066.2	9066.2	9066.2	9066.2	9066.2	9066.2
H-NS		15409.4	15409.4	15409.4	**15425.4**	**15425.4**	15409.4	15409.4	15409.4	15409.4	15409.4	15882.0	15409.4	15409.4	15409.4	15409.4	15409.4

Theoretical mass values (*m*/*z* [M+H]^+^) of possible biomarkers for discrimination of *E. coli* strains are shown. The database was constructed by validated *E. coli* strains available on public collections and three isolated strains. Groups A to P indicate the classification based on mass patterns. – in the group column indicates the O-antigen is not determined. – in MS column means the peaks are absent.

E. coli strains belong to the groups A to P are as bellows; A: O157-351, GTC 14545, GTC 14546, GTC 14552, O157-141, GTC 14513, GTC 14535, GTC 14536, GTC 14537, GTC 14544, GTC 14547, GTC 14551 and GTC 03904; B: O157-352; C: GTC 14550 and GTC 14553; D: GTC 14517, GTC 14507, GTC 14516, GTC 14538, GTC 14540, GTC 14549, GTC 14557 and GTC 14558; E: GTC 14515, GTC 14539 and GTC14548; F: GTC 14530, GTC 14601, GTC 14602, GTC 14603, JCM16574, NBRC 12062, NBRC 13168, NBRC 12734 and NBRC 3991; G: GTC 14518; H: GTC 14529; I: GTC 14559; J: NBRC 12713, ATCC 47076, NBRC 3301, NBRC 3972; K: NBRC 13893; L: NBRC 15034 and NBRC 14237; M: NBRC 13891; N: NBRC 3548; O: ATCC BAA-1743; P: JCM 16575.

**Figure 1:**
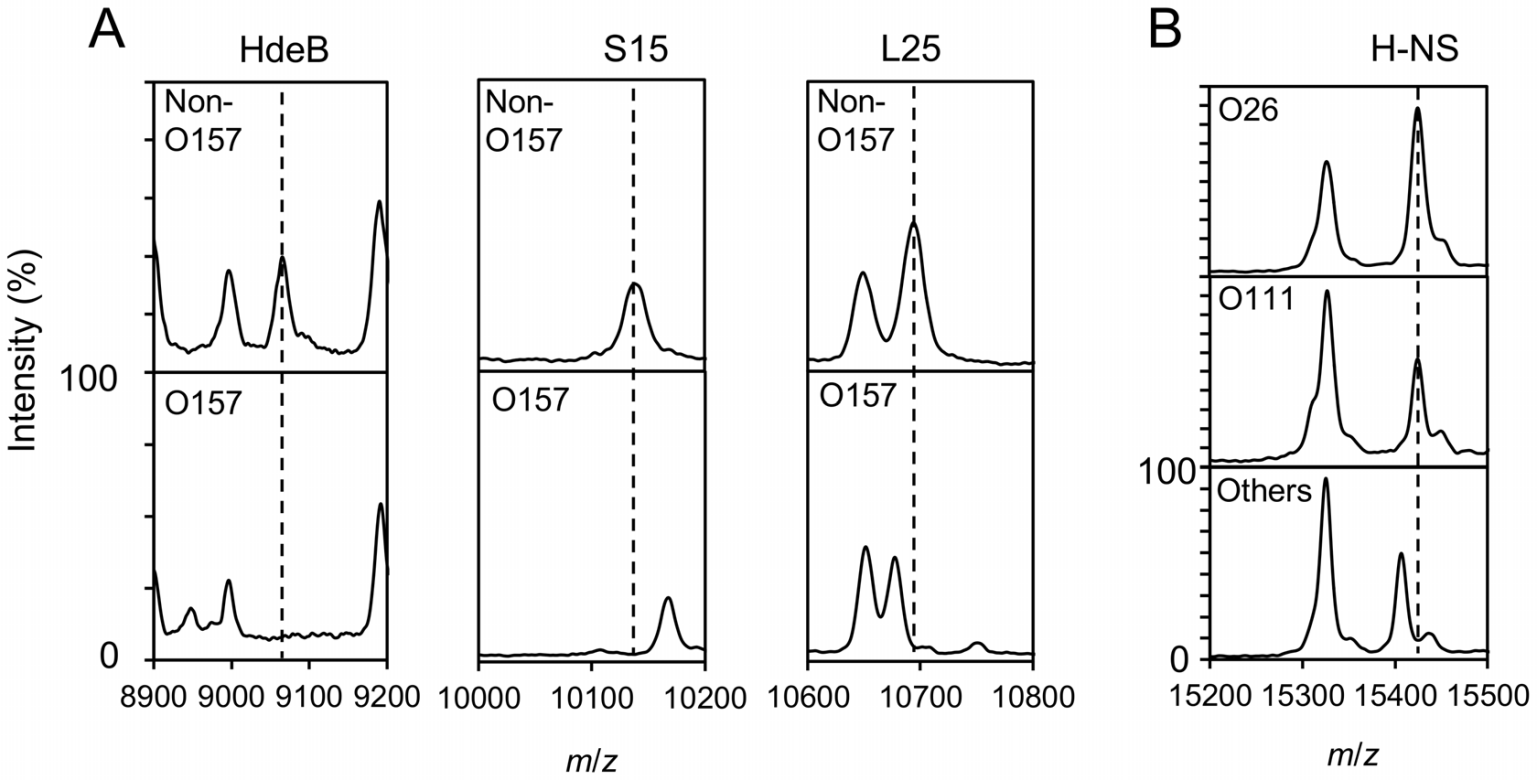
Typical mass spectra of four biomarker proteins in *E. coli*. A) MALDI mass spectra of non-EHEC *E. coli* strain NBRC12713 and EHEC *E. coli* strain O157 GTC 14513. Three biomarker peaks, HdeB (*m/z* 9066.2 [M+H]^+^), ribosomal protein S15 (*m/z* 10138.6/10166.6 [M+H]^+^) and L25 (*m/z* 10676.4/10694.4 [M+H]^+^), measured using sinapic acid as the matrix, are shown. B) Biomarker peak of DNA binding protein H-NS (*m/z* 15409.4/15425.4 [M+H]^+^) for strains O26, O111 and other *E. coli*.

Otherwise, unique and clear mass shifts of the ribosomal proteins S15 and L25 were observed specific in *E. coli* O157 compared with the other *E. coli* serovars (Fig. 1, Table 3). Sequence analysis revealed that a point mutation, A239G, on ribosomal protein S15 caused an amino acid residue change, Q80R, resulting in a MS shift of *m/z* 10138.6 to 10166.6. Similarly, the O157-specific mutation G150A in the gene encoding L25, resulting in an amino acid substitution, M50I, led to a mass shift of *m/z* 10694.4 to 10676.4. These two ribosomal proteins also showed mass shifts in the theoretical masses of *E. coli* strains GTC 14559, NBRC 15034, NBRC 14237, ATCC BAA-1743 and JCM16575 (group K, L, O and P in Table 3), although the differences were too small to distinguish in actual MALDI-TOF MS analysis. Exceptionally, two *E. coli* O157 strains, GTC 14550 and GTC 14553, showed the same mass patterns as most of the other strains except for the absence of *m/z* 9066.2.

To our knowledge, this is the first report that ribosomal proteins S15 and L25, H-NS would be important biomarkers for O157 in MALDI-TOF MS analysis, a finding overlooked by others [12]. The mass differences of ribosomal proteins greatly contribute to strain classification owing to their variability. The great abundance of these proteins in cells is also advantageous because their mass peaks are always detected as stable biomarkers under any analytical conditions (regardless of variables such as the method of sample preparation, the type of matrix or the MALDI system). In fact, the peak intensity and sharpness for proteins S15 and L25 in O157 serovars were sufficient to distinguish them from other *E. coli* serovars (Fig. 1). The same was possible using either sinapic acid or CHCA, whether the sample was a colony or a liquid extracted with formic acid (data not shown). Compared with the previously reported system that required time-consuming and complex sample preparation [12], our method is more applicable for routine MALDI-TOF MS analysis because it can be performed directly from a single colony.

The mass spectrum of the acid stress chaperone HdeB in non-EHEC strains was previously reported by Fagerquist *et al* [13]. Likewise we identified HdeB at *m/z* 9066.2 [M+H]^+^ in non-EHEC strains, and a loss of this peak was observed in all O157 serovars used in this study with complete reproducibility, as reported by Carter *et al* [15] (Fig. 1, Table 3). Sequence analysis of the *hdeB* gene confirmed that the putative start codon, ATG, had a point mutation (ATA) in all O157 strains, while in all other *E. coli* strains of other serovars ATG was observed. This strongly supported the suggestion that this mutation correlates to the lack of the HdeB peak in O157 strains [15].

The peak at *m/z* 6040 has been reported as a biomarker specifically present in O157 strains [12]. However, in our study, the intensity of the peak at *m/z* 6040 was too low to be detectable and in more than half of the O157 strains used for the mass database (namely GTC 14513, GTC 14535, GTC 14536, GTC 14537, GTC 14544, GTC 14547, GTC 14551 and GTC 03904) the peak was absent (data not shown), suggesting that the presence/absence of suspicious biomarker proteins of low intensity is insufficient as a method for discrimination at the strain or serovar level.

In this study, the identification of other prevalent EHEC strains (O26 and O111) was considered. O26 and O111 strains could be distinguished from other *E. coli* strains by the peak at *m*/*z* 15409.4/15425.4 [M+H]^+^ (Table 3, Fig. 1). From the sequence analysis, an amino acid change (A81S) in the DNA-binding protein H-NS in strains O26 and O111 was observed. A previous report had suggested that the protein corresponding to *m/z* 15409.4 [M+H]^+^ using K-12 strain (accession number P0ACF8) may be DNA-binding protein H-NS [18]; however, this required further confirmation. In our study, we first assigned the H-NS mass peaks and corresponding DNA sequences, and identified a specific mass shift in the H-NS protein in strains O26 and O111. Using the *S10*-GERMS method, in which the theoretical masses of biomarker proteins are confirmed, a valid and reliable mass database could be provided.

An additional peak at *m/z* 8326 [M+H]^+^ appeared to be another potential biomarker for *E. coli* classification (Table 3). Its mass was identical to the theoretical mass of hypothetical protein YdaQ in a TagIdent tool search (http://web.expasy.org/tagident/). PCR analysis of this gene was performed and a target band was detected in three out of the eight strains tested that did not show the peak at *m*/*z* 8326 (data not shown), suggesting that at least five strains may lack the *ydaQ* gene in their genome causing a loss of this peak in MALDI-TOF MS analysis. The expression level of YdaQ in the three strains possessing the corresponding gene may be low. Investigations into the identification of these biomarker proteins are now in progress.

### Effects of culture media

In species level discrimination by MALDI-TOF MS, growth condition often affect the expression pattern of proteins thus causes less reproducibility of mass spectra [19]. Here, colonies grown on various selective media were analyzed by MALDI-TOF MS and evaluated whether the important biomarker peaks HdeB, S15 and L25 for O157, and H-NS for O26 and O111 work well for their discrimination. As a result, ribosomal protein S15 and L25 whose mass shifts are characteristic to O157 were not affected by any growth medium in accordance with the previous report that the impact of growth conditions on ribosomal proteins were minimum [20]. Similarly the masses of DNA-binding protein H-NS were not affected by culture medium in any *E. coli* strain. On the other hand, mass intensity of HdeB in some strains of serovar O111 and O26 was decreased when grown on chromagar X-gal or VRBL, but nonetheless the peaks were enough to be detected in a default threshold. Therefore in the case of discrimination of O157, O26 and O111 from the others in colony directed MALDI-TOF MS analysis, the normal growth media and selective media such as desoxycholate agar, CT-SMAC, chromagar X-gal or VRBL will be available for the pre-selection of *E. coli*.

### Cluster analysis

**Figure 2:**
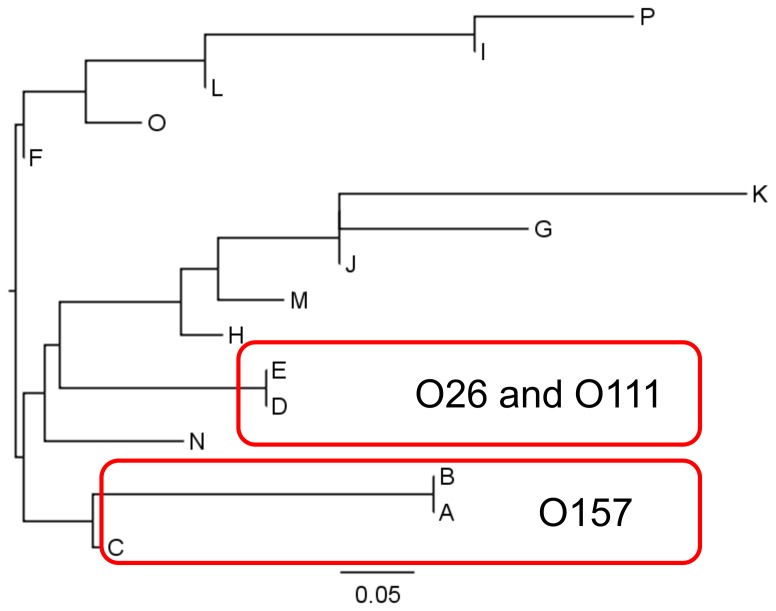
Cluster analysis for *E. coli* strains with selected biomarkers. Phylogenetic tree according to the binary graph made by actual analysis in Table 4 is shown. A to P indicate the *E. coli* groups classified by the theoretical mass patterns of biomarker protein peaks in Table 3.

All of the strains analyzed were correctly identified as *E. coli* by SARAMIS. Cluster analysis based on the theoretical mass database of 10 biomarker proteins listed in Table 3, in which the mass patterns were classified into groups A to P, was performed using the actually detected peaks in semi-automated MALDI-TOF MS analysis. As mentioned above, small mass shifts of around 1 Da in the S15 and L25 proteins observed in the theoretical database in group K, I, L, O and P in Table 1 were difficult to detect in actual MALDI-TOF MS analysis, and therefore differences in these proteins were not reflected in the cluster profiling summarized in Table 4. In a phylogenetic tree illustrated based on this profiling, all the O157 strains were correctly classified into groups A, B, or C namely the ‘O157 group’ (Fig. 2). In addition, strains O26 and O111 belonged to the same cluster, groups D and E, owing to a mass difference in the peak at *m*/*z* 15425.4, which was observed at *m*/*z* 15409.4 in the other *E. coli* strains tested (Fig. 1). Although high molecular weight proteins over 10000 Da are less detectable in many cases in MALDI-TOF MS [12], [17], they could be powerful biomarkers, as reported for *Salmonella* serovar identification [21]. To distinguish the small mass differences of S15 and L25 in group K, I, L, O and P from the others, MALDI-TOF MS analysis has mechanical limitation therefore MALDI-TOF/TOF-MS/MS analysis will be required to utilize such biomarkers in *E. coli*. Instead, the presence or absence of the *m*/*z* 8326 peak made it possible to apply more detailed grouping.

**Table 4:** Peak pattern of *E. coli* strains in actual MALDI-TOF MS analysis.

	Group of mass pattern															
	A	B	C	D	E	F	G	H	I	J	K	L	M	N	O	P
L23	1	1	1	1	1	1	2	1	1	1	1	1	1	1	1	1
L24	1	1	1	1	1	1	1	1	2	1	1	2	1	1	1	2
S14	1	1	1	1	1	1	1	1	1	1	1	1	1	2	1	1
L15	1	1	1	1	1	1	2	3	1	2	2	1	2	1	1	1
S11+Me	1	1	1	1	1	1	1	1	1	1	1	1	1	1	1	2
YdaQ	1	0	1	1	0	1	1	1	1	1	1	0	0	1	0	0
S15	2	2	1	1	1	1	1	1	1	1	1	1	1	1	1	1
L25	2	2	1	1	1	1	1	1	1	1	1	1	1	1	1	1
HdeB	0	0	0	1	1	1	1	1	1	1	1	1	1	1	1	1
H-NS	1	1	1	2	2	1	1	1	1	1	3	1	1	1	1	1

The actual mass of the peaks detected in MALDI-TOF MS has been replaced with number 1, 2 and 3 to represent the peak mass; 0 indicates no peak. Ribosomal protein S15 in group K and L25 in group I, L O and P were not distinguished from the others due to small mass differences in actual analysis with 500 ppm tolerance.

### Discrimination of isolated wild-type *E. coli* strains

The discrimination method proposed in this study was verified by performing blind tests using 12 *E. coli* strains (O157 and O26) named as jfrl 01–12, and 45 wild-type strains (O157, O26, O111 and O121) kindly provided from provided from APIPH (Table 1). Among total 41 O157 strains, all of them showed typical mass shifts of the ribosomal proteins S15 and L25 and an absence of the peak at *m/z* 9066.2 with MALDI-TOF MS analysis. The ribosomal protein L23 and L24 were eliminated from the biomarkers because their mass peaks were not clear. Nevertheless they were correctly classified into the O157 group as categorized in Table 3 and Fig. 2. In detail, 39 strains were classified into group A and jfrl 01 and 07 were classified into group B due to a loss of the *m/z* 8326 peak. This result indicates that the variety of mass patterns in our database may be sufficient for serovar level discrimination of wild-type O157 strains regardless of the place or date of isolation. It should be noted that among the genome or partial sequence available strains of *E. coli* O157 (total 126 strains in the NCBI database), 119 (94.4%) strains including Sakai, FRIK2000, EC4206, EC4045, EC4196, EC4076, EC4113, EC4486, EC869, EC4501, EC508, EC4024, FRIK966, EC4115, EC4401, EC4486, EC4501, TW14588, TW14359, EDL933 and EC4042, have the same theoretical mass patterns for the biomarker proteins S15 and L25 as group A, that is typical ‘O157 group’ in our experiments. Only 7 strains in the database, namely G5101, 493–89, H 2687, LSU-61, 2010C-4979C1 and 98–3133, show the same masses of the other *E. coli* strains as classified in group C type O157 in Table 3. These findings suggest that a mass shift of ribosomal proteins S15 and L25 is common in most of O157 strains in the database, indicating that our discrimination approach that focuses on the mass shifts of S15 and L25 with the combination of a loss of HdeB peak, could be universally applied for O157 strain discrimination worldwide.

Similarly 11 O26 strains and 4 O111 strains for blind test were correctly classified into group E in Table 3 and Fig. 2 due to specific masses of H-NS. Although we could not isolate another O26 and O111 samples, the sequence available O26 strains in database (strain 11368, CVM9942, CVM10026, CVM10224, CVM10021, CVM9952, CVM10030, CFSAN001629, 2010C-4347, 2010C-4788, 05-3646, 06-3464, 03-3500, 2010C-4430, 2010C-4819, 2010C-4834, 2010C-5028, 2011C-3270, 2010EL-1699, 2011C-3387, 2011C-3282, 2011C-3506, 2011C-3655, 2009C-3612, 2009C-3689, 2009C-3996, 2009C-4760, 2009C-4826, 2010C-3051, 2010C-3871, 2010C-3472, 2010C-3902, 2010C-4244 and 2009C-4747) and O111 strains (strain 11128, CVM9534, CVM9574, CVM9570, CVM9545, CVM9602, CVM9634, CVM9455, CVM9553, CFSAN001632, CFSAN001630, 2010C-4221, 2010C-3977, 2010C-4086, 08-4487, 2011C-3632, 2011C-3679, 2011C-3573, 2011C-3362, 2011C-3170, 2010C-4818, 2010C-4799, 2010C-4746, 2010C-4735, 2010C-4715, 2010C-4622, 2010C-4592, 04-3211, 03-3484, F6627, K6723, K6728, K6722, K6890, K6895, K6897, K6904, K6898, K6908, K6915, 2009C-4006, 2009C-4052, 2010C-3053, 2009EL-2169, 2009C-4126 and 2011C-3453) have the same theoretical mass values for DNA binding protein H-NS as group E, namely ‘O26 and O111 group’. Therefore our findings for O26 and O111 will be also applicable for the isolates regardless of the date or place. A remaining wild-type strain, O121 was classified into group F.

To define O157, O26 and O111 from the others in a routine MALDI-TOF MS analysis in laboratory, masses of four selected biomarkers must be characteristic to these serovars. Although we have tested more than 11 different serovars in the present study for the construction of database and confirmed that at least no other serovars showed the same mass patterns as either O157, O26 or O111 in this experimental scale (Table 1, Table 3) and the biomarkers were effective in the blind test using 57 wild-type strains including O121, the examination with wider variety of serovars is desired. Since it was difficult to obtain *E. coli* strains with various serovars, the probability of our database was validated *in silico* by checking the theoretical masses of biomarkers in various types of *E. coli* serovars. Out of more than thousands of *E. coli* strains available in the NCBI database, the theoretical masses of ribosomal protein S15 and L25 in all non-O157 *E. coli* strains were calculated as *m/z* 10138.6 and 10694.4, respectively. They are completely equal to that of group C to P shown in Table 3, namely ‘non-O157 group’, indicating the database constructed in this study will work well for screening of O157 from various types of serovars.

On the other hand, the specific biomarker, H-NS (*m/z* 15425.4 [M+H]^+^) for O26 and O111 were observed in another few strains, O118 (strain 08-3651, 06-3612, 06-3256, 2009C-4446, 07-4255), O69 (strain 07-4281, 06-3325, 08-4661, 2009C-3601, 07-3763), O123 (strain 2009C-3307) and O103 (strain 2010C-3214). As they may be classified into the same group with O26 and O111 in our discrimination system using H-NS as biomarker, the extra biomarkers will be required for more detailed identification. Nevertheless it is promising that candidates for serovar O26 and O111 could be found in our system.

**Figure 3:**
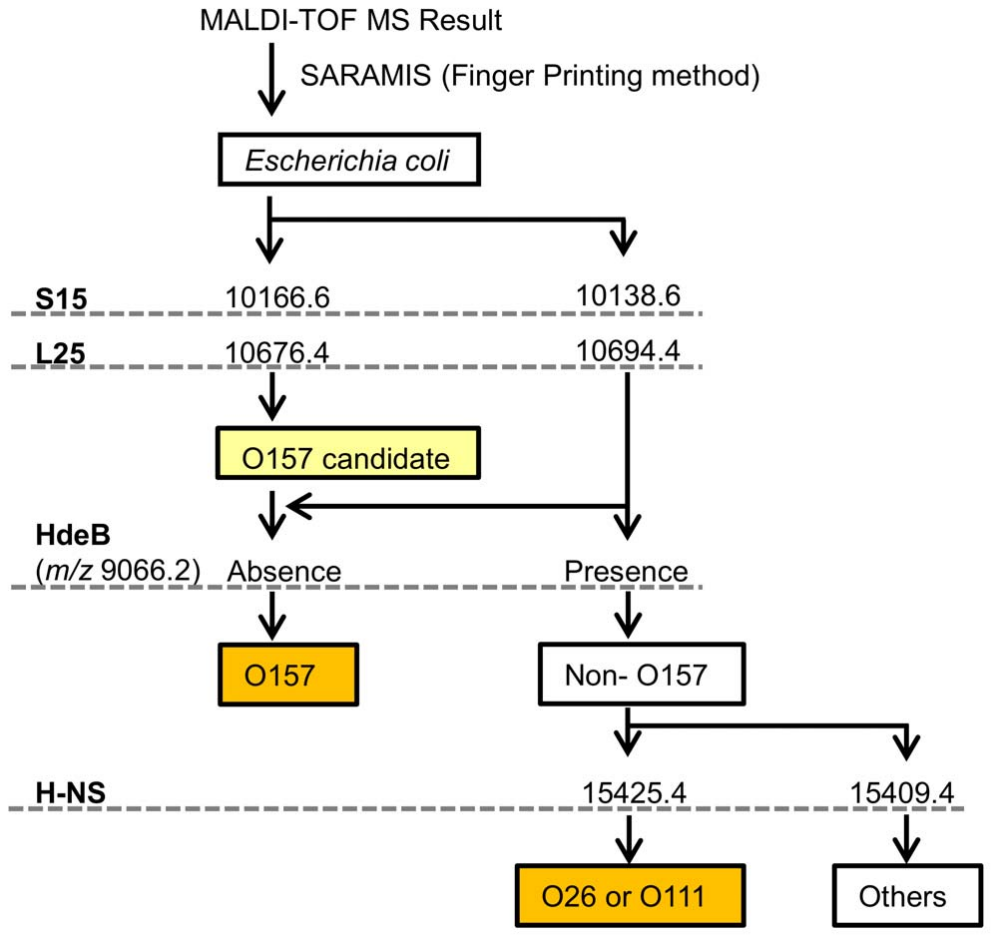
Strategy for distinguishing between *E. coli* strains O157, O26, O111 and the others using four biomarker peaks in MALDI-TOF MS.

The major EHEC serovars O157, O26 and O111 present a great risk for human life, and therefore not only will the rapid discrimination of this strain from other *E. coli* strains aid diagnostics, but it is also vital in ensuring clinical security and food safety. We propose a possible strategy for the effective discrimination of strains O157, O26 and O111 using specific four biomarkers by MALDI-TOF MS as shown in Fig. 3. Our *S10*-GERMS-based discrimination method uses the arbitrary selected masses of established biomarkers that are confirmed from the approaches of both genomics and proteomics. For automated processing and clustering of the data generated by MALDI-TOF MS, the analytical software ‘Strain Solution’ (Shimadzu, Kyoto, Japan) could be employed to realize the *S10*-GERMS approach. Our discrimination method will be an important screening tool clinically and in the dairy industry to ensure food safety.
